# High-level de novo biosynthesis of glycosylated zeaxanthin and astaxanthin in *Escherichia coli*

**DOI:** 10.1186/s40643-021-00415-0

**Published:** 2021-07-29

**Authors:** Xixian Chen, Xiaohui Lim, Aurélie Bouin, Thomas Lautier, Congqiang Zhang

**Affiliations:** 1 Singapore Institute of Food and Biotechnology Innovation (SIFBI), Agency for Science, Technology and Research (A*STAR), Proteos level 4, Nanos, 138673 Singapore; 2 TBI, Université de Toulouse, CNRS, INRAE, INSA, Toulouse, France

**Keywords:** Carotenoids, Metabolic engineering, Synthetic biology, Glycosylation, Glucosyltransferase, Zeaxanthin, Astaxanthin, UPD-glucose

## Abstract

Because of wide applications in food, feed, pharmaceutical and cosmetic industries, the carotenoid market is growing rapidly. Most carotenoids are hydrophobic, which limits their bioavailability. Glycosylation is a natural route that substantially increases the water solubility, as well as the bioavailability, photostability and biological activities of carotenoids. Here, we report metabolic engineering efforts (e.g., promoter and RBS engineering, optimization of carbon sources and supplementation of bottleneck genes) to produce glycosylated carotenoids in *Escherichia coli*. By fine-tuning the carotenoid-biosynthetic genes (*crtX*, *crtZ* and *crtY*), our strain produced up to 47.2 mg/L (~ 11,670 ppm) of zeaxanthin glucosides, ~ 78% of the total carotenoids produced. In another construct with mevalonate, astaxanthin pathway and *crtX* genes, the strain produced a mixture of carotenoid glucosides including astaxanthin and adonixanthin glucosides with a total yield of 8.1 mg/L (1774 ppm). Our work demonstrated a proof-of-concept study for the microbial biosynthesis of glycosylated carotenoids.

**Supplementary Information:**

The online version contains supplementary material available at 10.1186/s40643-021-00415-0.

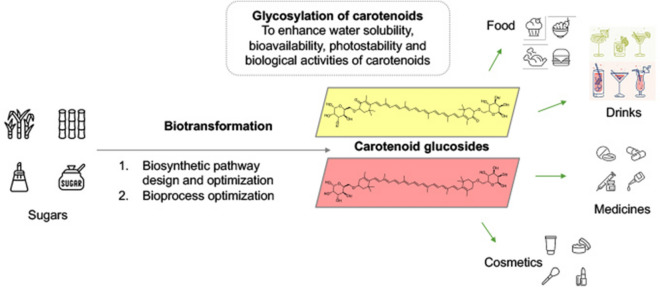

## Introduction

Carotenoids ( > 1100) are natural pigments widely distributed in plants, animals, algae and microbes (Yabuzaki 2017; Zhang 2018). The structures of carotenoids typically consist of an electron-rich polyene chain with nine or more conjugated double bonds. This unique feature contributes primarily to their photoprotection and light-harvesting property, antioxidant activities to quench free radicals and singlet oxygen, and vivid colors (Sandmann 2019). Carotenoids function as photosynthesis and photoprotection agents in photosynthetic organisms (e.g., plants and algae) and protect non-photosynthetic organisms (e.g., bacteria, archaea and fungi) from photooxidative damages (Hashimoto et al. 2016). Carotenoids also serve as structural molecules by integrating in lipid membranes, hence, modulating membrane fluidity (Richter et al. 2015). Because of these properties, especially for the pigment and health benefits, carotenoids have various applications in food, feed, nutraceutical and pharmaceutical industries, and the industrial demand is growing rapidly. For example, the global market of astaxanthin is projected to reach $2.57 billion worldwide by 2025 (Zhang et al. 2020).

However, most natural carotenoids are lipophilic and hardly soluble in water. The hydrophobicity of carotenoids limits their application in medicine and food where enhanced water dispensability is required to facilitate their effective uptake or use (Dembitsky 2005; Hada et al. 2012). Therefore, several attempts, mainly chemical approaches (e.g., converting carotenoids to salts of carotenoid esters, or forming carotenoid–cyclodextrin complex), have been made to increase the carotenoid hydrophilicity (Hada et al. 2012). Alternatively, glycosylation is an excellent natural way to increase carotenoid solubility. In nature, a large number of hydrophobic natural products (e.g., lipids and terpenes) are glycosylated into more water-soluble products by glycosyltransferases (Elshahawi et al. 2015). In fact, water-soluble carotenoids, although rare, are present in nature, such as crocins (or glycosyl polyene esters) in saffron (Dembitsky 2005). In addition, several other glycosylated carotenoids are uncovered in various microbes, such as zeaxanthin glucoside (Misawa et al. 1990), astaxanthin glucoside (Yokoyama et al. 1998), adonixanthin-β-D-glucoside (Yokoyama et al. 1995), sioxanthin (Richter et al. 2015) and a C50 decaprenoxanthin diglucoside (Krubasik et al. 2001).

Natural metabolites are typically produced meaningfully with biological functions for host living organisms. Primary metabolites are synthesized to support their growth and development. Secondary metabolites typically increase the competitiveness of the organism within its environment. Likewise, glycosylated carotenoids should have meaningful functions for their hosts. It is reported that glycosylated carotenoids play important roles in maintaining cell wall structure and their localization stabilizes the thylakoid membrane in cyanobacteria where the glycosyl moiety serves as a binding motif that enables the proper folding and stacking of the thylakoid membrane (Mohamed et al. 2005). The first bacterial gene that encodes the enzyme to catalyze carotenoid glycosylation was identified in *Pantoea ananatis* (previously as *Erwinia uredovora*) (Misawa et al. 1990) and it was reported that glycosylation can alter carotenoid deposition in plants (Wurtzel 2019). As a phytopathogen, this might contribute to the virulence of *P. ananatis* with host plant cells. Moreover, carotenoid glucosides contribute to the heat resistance of the *Thermus* species, and hence, are also named thermoxanthins (Hada et al. 2012). As for commercial applications, apart from improved water solubility (e.g., the solubility of zeaxanthin, zeaxanthin mono- and diglucosides are 12.6, 100 and 800 ppm in water, respectively (Hundle et al. 1992)), glycosylation of carotenoids also leads to structural diversity and several other benefits, such as increased bioavailability and efficacy as food supplements and medicines, and improved photostability (Polyakov et al. 2009) and biological activities (e.g., antioxidant activity) of carotenoids (Matsushita et al. 2000). It is proposed that the increase in antioxidant activities is not from their intrinsic ability of additional glucosides to scavenge free radicals, but arises from the enhanced affinity with singlet oxygen, the location and orientation in cells (Choi et al. 2013; Matsushita et al. 2000).

Carotenoids are glycosylated by glycosyltransferases (GTs), which is a large enzyme family. GTs typically catalyze a hydroxyl or carboxyl group of lipophilic substrates as the substituent moiety for glycosylation. For carotenoid glycosylation, the hydroxyl group is the commonest substituent moiety, and the carotenoid GTs belong to GT family 1 or GT1. Uridine diphosphate-α-D-glucose (UDP-glucose) is the most abundant sugar donor to carotenoid glycosylation. In addition, other sugars such as L-rhamnose, L-fucose, D-xylose and L-quinovose can also be recruited especially in cyanobacteria (Choi et al. 2013).

To date, only a couple of studies have demonstrated the biosynthesis of carotenoid glucosides in *Escherichia coli* and in several natural microbial producers (Choi et al. 2013; Misawa et al. 1990; Yokoyama et al. 1995,1998). However, these studies only produced detectable amount of carotenoid glucosides and were far from the minimal requirement for industrial applications. Here, using the zeaxanthin glucosyltransferase (ZGT, the gene *crtX*, UniProt ID D4GFK6) from *P. ananatis*, we have constructed a 14- and 15-gene pathway in *E. coli* to synthesize various carotenoid glucosides, such as zeaxanthin D-glucoside (yellow) and astaxanthin D-glucoside (red). The carotenoid yields have been improved by rational metabolic engineering approaches and bioprocess optimization.

## Results

### The pathway design for glycosylated carotenoids

**Fig. 1:**
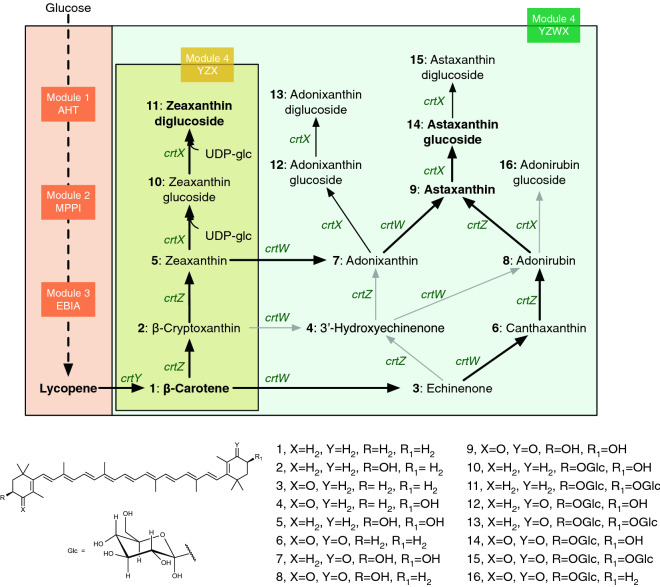
Biosynthetic pathway of carotenoid glucosides. The biosynthetic pathway: module 1 AHT, including *atoB*, *hmgS* and *thmgR*; module 2 MPPI, including *mevk*, *pmk*, *pmd* and *idi*; module 3 EBIA, including *crtEBI* and *ispA* (Zhang et al. 2018); and module 4 YZX or YZWX, including *crtYZX* or *crtYZWX*. Dashed arrow indicates multiple enzymatic steps. The glycosylation of all carotenoids required UDP-glucose (UDP-glc), here we only used zeaxanthin glucosides as representatives. The genes expressed encode the following enzymes: *crtY*, lycopene beta-cyclase; *crtW*, β-carotene ketolase; *crtZ*, β-carotene hydroxylase; *crtX*, zeaxanthin glucosyltransferase (ZGT). Thicker and thinner arrows represent the higher and lower carbon flux, respectively; gray arrows represent that the metabolites (e.g., β-cryptoxanthin-β-D-glucoside and 3′-hydroxyechinenone-β-D-glucoside) were not detected in our strains

The metabolic pathway for glycosylated carotenoids was designed on top of our previous optimized astaxanthin strain (Zhang et al. 2018). Briefly, the mevalonate pathway genes were cloned into the modules 1 (AHT, the genes *atoB*, *hmgB* and truncated *hmgR*) and 2 (MPPI, the genes *mevk*, *pmk*, *pmd* and *idi*) and the lycopene pathway genes (*crtEBI* and *ispA*) were located in module 3 (EBIA). The last module (module 4, YZX or YZWX) consists of the genes to produce zeaxanthin glucosides (*crtY, crtZ*, *and crtX*) or to produce astaxanthin glucosides (*crtY, crtZ, crtW*, and *crtX*) (Fig. 1). All the modules were controlled by T7 and its variants (e.g., TM1, TM2 and TM3) and induced by isopropyl β-d-1-thiogalactopyranoside (IPTG) (Zhang et al. 2015). This modular arrangement provides the flexibility to balance the global pathways (14–15 genes) and to fine tune the local pathways (e.g., module 4). In addition, as the module 4 controls the cyclization (*crtY*), hydroxylation (*crtZ*), ketolation (*crtW*), and glycosylation (*crtX*) of carotenoids, it is relatively simple to switch from one carotenoid (e.g., using *crtYZ* to produce zeaxanthin) to another one (e.g., using *crtYZWX* to astaxanthin glucoside) without modifying the upstream pathways genes.

### The production of glycosylated zeaxanthin

**Fig. 2:**
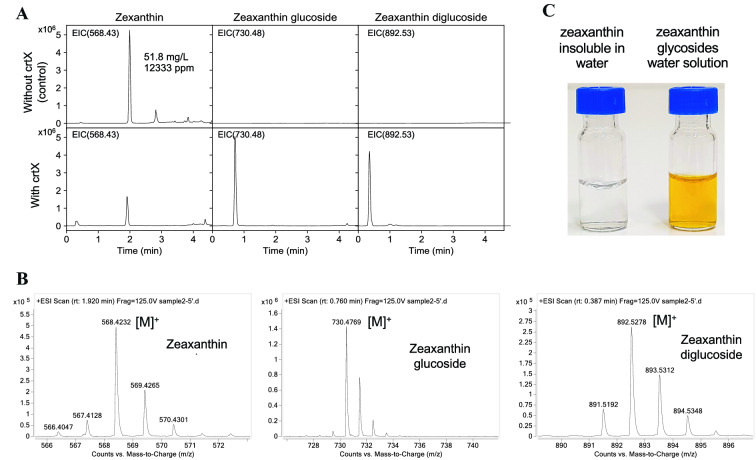
Production of zeaxanthin glucosides. **A** LC/MS chromatograms of zeaxanthin strains with and without the expression of *crtX*. **B** Mass spectra of zeaxanthin and its glucosides. **C** The water solutions of zeaxanthin and zeaxanthin glucosides

Before we produce glycosylated zeaxanthin, we first optimized a strain that produces zeaxanthin (the last module contains *crtY* and *crtZ*, or module YZ), the combination of TM3-AHT, TM2-MPPI, TM2-EBIA and T7-YZ resulted in the best production of zeaxanthin (~ 12,000 ppm or 51.8 mg/L). On top of this strain with the same modules 1–3, we introduced module 4 (YZX) to demonstrate the capability to produce zeaxanthin glucoside. We developed a LC–TOF-MS method to detect the carotenoids and their glucosides (summary in Table 1). In the constructed strain with *crtX*, we managed to detect five carotenoids: lycopene, β-carotene, zeaxanthin, zeaxanthin-β-D-glucoside and zeaxanthin-β-D-diglucoside (Additional file 1: Figure S1); whereas, the control strain without *crtX* did not produce either glycosylated zeaxanthin (Fig. 2A). The intermediate β-cryptoxanthin was not detected in either strain. The LC chromatograms and mass spectra for zeaxanthin (m/z 568.428, Table 1), zeaxanthin-β-D-glucoside (m/z 730.481) and zeaxanthin-β-D-diglucoside (m/z 892.534) are shown in Figs. 2A, B. In addition, we also purified some zeaxanthin glucosides from the strain with *crtX* and obtained a yellow aqueous solution (~ 30 mg/L). In contrast, zeaxanthin barely dissolves in water leading to a transparent water solution (Fig. 2C).

**Table 1:** Carotenoid information

No	Compound	Chemical structures	RT (min)	Chemical formula	Monoisotopic mass (m/z)
1	β-carotene		8.84	C_40_H_56_	536.438
2	Lycopene		7.37	C_40_H_56_	536.438
3	Echinenone		5.07	C_40_H_54_O	550.417
4	β-cryptoxanthin		4.53	C_40_H_56_O	552.433
5	Canthaxanthin		3.38	C_40_H_52_O_2_	564.397
6	Zeaxanthin		2.11	C_40_H_56_O_2_	568.428
7	Adonirubin		1.91	C_40_H_52_O_3_	580.392
8	Adonixanthin		1.35	C_40_H_54_O_3_	582.407
9	Astaxanthin		0.98	C_40_H_52_O_4_	596.387
10	Zeaxanthin-β-D-glucoside		0.78	C_46_H_66_O_7_	730.481
11	Adonirubin-β-D-glucoside		0.81	C_46_H_62_O_8_	742.444
12	Adonixanthin-β-D-glucoside		0.51	C_46_H_64_O_8_	744.460
13	Astaxanthin-β-D-glucoside		0.43	C_46_H_62_O_9_	758.439
14	Zeaxanthin-β-D-diglucoside		0.37	C_52_H_76_O_12_	892.534
15	Adonixanthin-β-D-diglucoside		0.31	C_52_H_74_O_13_	906.513
16	Astaxanthin-β-D-diglucoside		0.30	C_52_H_72_O_14_	920.492

### Optimization of glycosylation of zeaxanthin

**Fig. 3:**
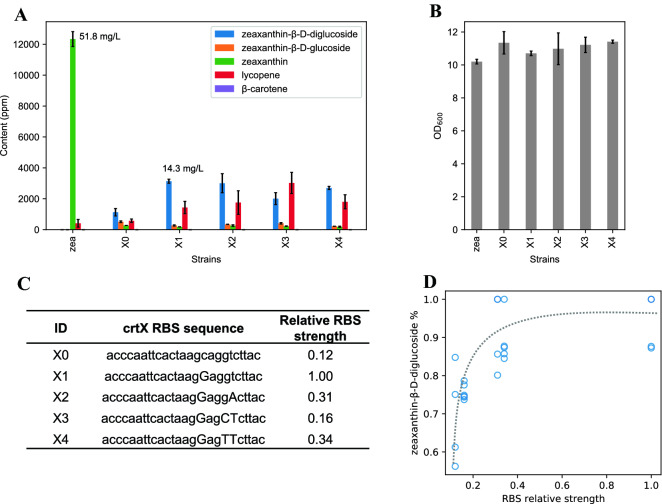
Tuning the translation of zeaxanthin glucosyltransferase. **A** Carotenoid contents of zeaxanthin glucoside strains. ‘zea’ strain is the parental zeaxanthin strain without expressing *crtX*. **B** OD_600_ of different strains. Error bars, mean ± s.d., *n* = 2 or 3. **C** Different RBSs used for *crtX* and their relative strengths. **D** Correlation between the glycosylation efficiency of zeaxanthin and the RBS strength of *crtX*. The glycosylation efficiency is defined as the percentage of zeaxanthin diglucoside yield to the total yield of zeaxanthin and its two glucosides

In our first design strain X0, the glycosylation of zeaxanthin was incomplete: ~ 26.8% of monoglycosylated and 59.0% of diglycosylated (here the percentage was calculated by normalizing to the total yield of zeaxanthin and its two glucosides) and 14.2% of zeaxanthin remained unglycosylated (Fig. 3A, B). We hypothesized that glycosylation of zeaxanthin could be limited by insufficient activity of ZGT. To test it, we re-designed another four ribosomal-binding sites (RBSs) of *crtX* which have relatively higher translational efficiencies than the initial RBS in strain X0 (Fig. 3C). Indeed, we observed that using stronger RBS for ZGT (*crtX*) led to higher glycosylation of zeaxanthin (Fig. 3A, D). Strain X1 had the strongest RBS and produced the highest amount of zeaxanthin-β-D-diglucoside (~ 3139 ppm and ~ 87.4% of total zeaxanthin and its glucosides). We attempted to correlate RBS strengths to zeaxanthin-β-D-diglucoside production. Zeaxanthin-β-D-diglucoside produced appears to reach a saturated percentage when RBS relative strength was higher than 0.3 (Fig. 3D). It was noteworthy that the total yield of carotenoids in zeaxanthin glucoside strains (X0–X4) was about 50–80% lower than that of parental zeaxanthin strain (zea).

**Fig. 4:**
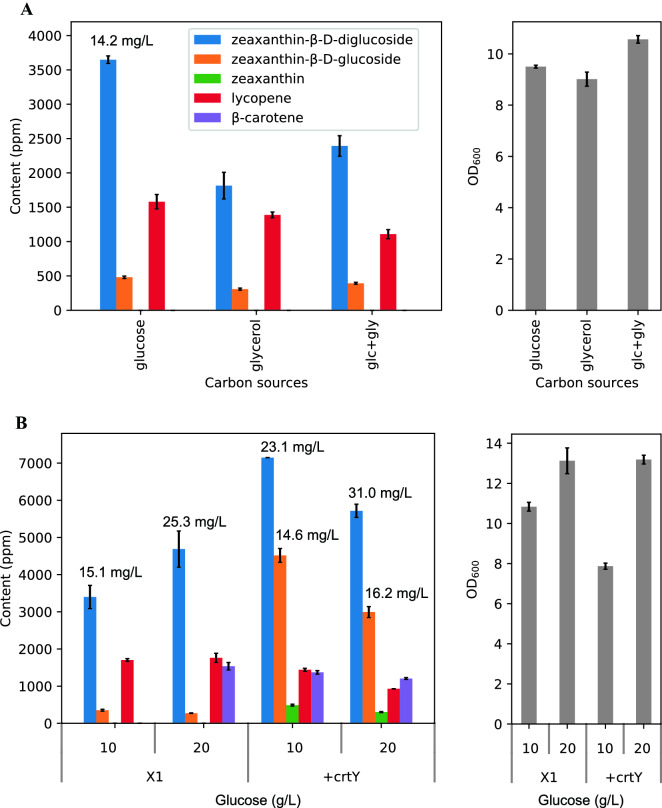
The effects of carbon sources on the production of zeaxanthin glucosides. **A** Carotenoid contents and OD_600_ of strain X1 by comparison of different carbon sources: 10 g/L glucose, 10 g/L glycerol and their mixture, 5 g/L glucose + 5 g/L glycerol (glc + gly). **B** Carotenoid contents and OD_600_ of strains X1 and “ + crtY” by optimizing the concentrations of glucose and introduction of additional copies of *crtY*. Error bars, mean ± s.d., *n* = 2

Next, we evaluated the effect of different carbon sources on the biosynthesis of zeaxanthin glucosides. As an abundant and inexpensive carbon source, we chose glucose and hypothesized that glucose might be advantageous to supply additional UDP-glucose, which is the key cofactor for carotenoid glycosylation. UDP-glucose can be produced from glucose with three enzymes: *glk*: glucokinase, *pgm*: phosphoglucomutase, galU: UDP-glucose pyrophosphorylase (Mao et al. 2006; Shrestha et al. 2019). In addition, we also chose glycerol as it is inexpensive and was reported to favor carotenoid production (Zhang et al. 2013). For X1 strain, the glucose supplementation (10 g/L) led to higher production of zeaxanthin glucosides (~ 3650 ppm) than the supplementation of 10 g/L of glycerol or the mixture of glucose (5 g/L) and glycerol (5 g/L) (Fig. 4A). Subsequently, we increased the amount of supplemented glucose from 10 to 20 g/L, the yield of zeaxanthin diglucoside was further increased from ~ 3400 (or 15.1 mg/L) to ~ 4690 ppm (or 25.3 mg/L). At the same time, OD_600_ was also increased from 10.8 to 13.1 (Fig. 4B). Of the total carotenoids produced including lycopene and β-carotene, zeaxanthin glucosides reached about 64% in X1 strain.

In addition, we also observed that lycopene was accumulated as the main intermediate carotenoid for all the strains and conditions in Figs. 3A, 4A. We hypothesized that the accumulation of lycopene could arise from the insufficient activity of lycopene cyclase (or *crtY*, Fig. 1). Indeed, the introduction of extra copies of *crtY* (“ + crtY” strain) significantly boosted zeaxanthin diglucoside yield from 3400 to 7150 ppm (or 23.1 mg/L) and zeaxanthin glucoside yield from 350 to 4520 ppm (14.6 mg/L) in the medium supplemented with 10 g/L glucose (Fig. 4B). Furthermore, for the “ + crtY” strain, the titres of zeaxanthin diglucoside and glucoside were further increased to 31.0 and 16.3 mg/L, respectively, as the supplemented glucose was increased from 10 to 20 g/L (Fig. 4B). Lastly, the yields of zeaxanthin glucosides of “ + crtY” strain were about 78% of that of total carotenoids produced.

### Distribution of carotenoids in *E. coli* cells

**Fig. 5:**
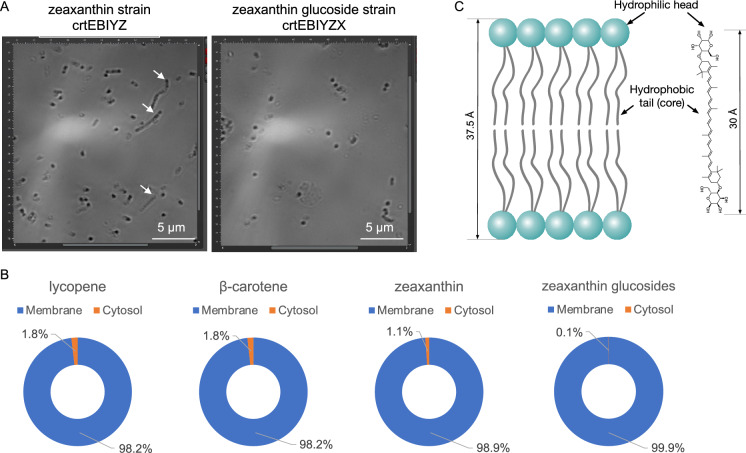
Structural similarity between membrane and carotenoid diglucosides and its biological benefits. **A** Comparison between zeaxanthin and zeaxanthin glucosides strains. **B** Carotenoid distribution between cytosol and membrane. **C** Structural similarity between phospholipid bilayers and zeaxanthin diglucoside and their dimensions

While studying the zeaxanthin glucoside strain, we observed that some cells of zeaxanthin production strain were longer than others in microscopes (Fig. 5A). In comparison, there were no elongated cells for zeaxanthin glucoside production strain. We wondered if the cell shape difference was attributed to the higher hydrophilicity of zeaxanthin glucosides so that most zeaxanthin glucosides may be distributed in cytosol. To test the hypothesis, we analyzed the distribution of carotenoids between cytosol and membrane. Unexpectedly, it was found that all the four carotenoids (lycopene, β-carotene, zeaxanthin and zeaxanthin glucosides) were predominantly localized in membrane (Fig. 5B). Less than 2% of them were present in cytosol. In addition, less zeaxanthin glucosides (0.08%) was distributed in cytosol as compared to zeaxanthin (1.13%). Our data supported the notion that zeaxanthin and its glucosides might have higher affinity with membrane than cytosol. Structurally, the glucoside and carotene of carotenoid glucosides resemble the hydrophilic head and the hydrophobic tail of phospholipid bilayers, respectively; also, the dimension of bilayer inner membrane (37.5 ± 0.5 Å) (Mitra et al. 2004) is close to that of zeaxanthin diglucoside (~ 30 Å) (Fig. 5B). Carotenoid glucosides are reported to be clustered in rigid patches and such local rigidity can protect the membrane integrity under internal or external stress (e.g., oxidative and extreme temperature) (Mohamed et al. 2005). This might attribute to cell shape difference between zeaxanthin and zeaxanthin glucoside-producing cells, and further study is warranted to explore the mechanism.

### The production of glycosylated astaxanthin

**Fig. 6:**
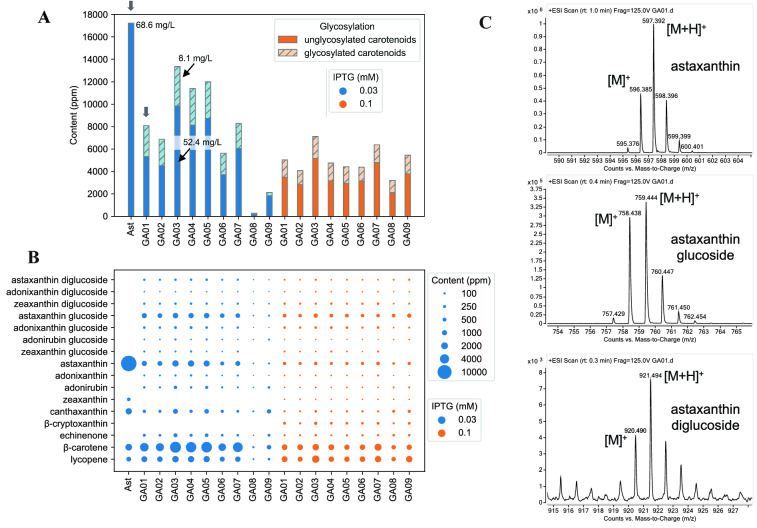
Production of astaxanthin glucosides and other carotenoids. **A** The content sums of glycosylated and unglycosylated carotenoids in different strains. The arrows refer to the two control strains (‘Ast’ and ‘GA01’). **B** Carotenoid contents produced in different strains. Blue: 0.03 mM IPTG; orange: 0.1 mM IPTG. ‘Ast’ strain is the parental astaxanthin strain without expressing *crtX*. ‘GA01’ is the control strain with the highest RBS strength of *crtZ*. **C** Mass spectra of astaxanthin and its glucosides

After demonstrating our design was working for zeaxanthin glycosylation, we further tested the other design with module YZWX to produce astaxanthin glucosides. With the addition of the gene *crtX* in one of our best astaxanthin producer strains (Ast strain, Fig. 6A, B) (Zhang et al. 2018), we tested the astaxanthin glycosylation capability (the resulting strain was named GA01). Overall, seven carotenoid glucosides are detected in GA01: zeaxanthin-β-D-glucoside, adonirubin-β-D-glucoside (m/z 742.444), adonixanthin-β-D-glucoside (m/z 744.460), astaxanthin-β-D-glucoside (m/z 758.439), zeaxanthin-β-D-diglucoside, adonixanthin-β-D-diglucoside (m/z 906.513) and astaxanthin-β-D-diglucoside (m/z 920.492, Fig. 6A, B, Table 1, mass spectra in Figs. 6C and Additional file 1: Figure S2, and LC chromatograms in Additional file 1: Figure S3 and S4). Among them, astaxanthin-β-D-glucoside was the main glycosylated product with a yield of 4.51 mg/L (968 ppm), about 68% of total carotenoid glucosides. In addition, about 4.82 mg/L astaxanthin (1035 ppm) was not glycosylated and larger amount of β-carotene (16.0 mg/L, 3426 ppm) remained in GA01 strain. Furthermore, we observed that the introduction of *crtX* resulted in a 54% decrease of the total carotenoid yields in GA01 strain, as compared to its parental Ast strain (Fig. 6A), which might be due to the overall perturbation to the mevalonate and carotenoid pathway carbon fluxes or feedback regulations.

Here, we would like to highlight that in the our conditions (ESI mode, with water, methanol and acetonitrile as mobile phase), the detected molecular-related ions of carotenoids had two kinds: [M]^+.^ and [M + H]^+^. Different carotenoids can have different ratios of these two ion species. For zeaxanthin and its glucosides, [M]^+.^ was predominant (Fig. 2B), while for astaxanthin and its glucosides, both [M]^+.^ and [M + H]^+^ co-existed (Fig. 5C). This phenomenon was observed previously (Rivera et al. 2014), and it was found that the high polyene conjugation, the presence of oxygen in carotenoids and solvent system have a strong impact on the formation and stability of molecular ion species (Rivera et al. 2014).

### Optimization of glycosylation of astaxanthin

Moreover, the higher IPTG concentration reduced the total yield of glycosylated carotenoids from 6.61 to 3.60 mg/L (1418 to 799 ppm) and non-glycosylated (or aglycones) carotenoids from 24.8 to 15.7 mg/L (5320 to 3485 ppm, Fig. 6A), possibly because IPTG perturbed the whole biosynthetic pathway where all the genes were controlled by T7 promoter variants and/or it promoted a competition between CrtZ and CrtW with intermediate accumulation (Fig. 1). It has been observed that the translational efficiency of the β-carotene hydroxylase (*crtZ*) is more crucial than that of β-carotene ketolase (*crtW*) on astaxanthin production (Zhang et al. 2018). Therefore, we used nine different ribosomal-binding sites (RBSs, Additional file 1: Table S1) covering from 1 to 100% of translational efficiencies (the strains were named G01–09, translational efficiencies were normalized to that of strain GA01, the strongest among them) to optimize the production of glycosylated carotenoids, especially glycosylated astaxanthin.

Essentially, GA01–09 were strains with the same design except for the different RBSs of *crtZ* (Additional file 1: Table S1). Indeed, the RBS had marked effects on the carotenoid production and distribution (Fig. 6A, B, Additional file 1: Figure S5). For GA08 and GA09, the total carotenoid yields were very low, below 10 mg/L (< 2000 ppm), and the carotenoid glucosides were also very low, below 0.4 mg/L (< 100 ppm). GA01 and GA02 had the highest glycosylation efficiency (~ 21%, Fig. 6A), but with relatively lower total carotenoid yields as compared to GA03, GA04 and GA05. Surprisingly, GA03, with a relatively weaker RBS (Additional file 1: Table S1), had the highest yield of total carotenoids (11,623 ppm) and total glycosylated carotenoids (1774 ppm). Similar to GA01, strains GA02–07 had lower yields of carotenoids (including glycosylated carotenoids) when IPTG concentrations increased from 0.03 to 0.1 mM. In contrast, strains GA08–09 had higher yields when IPTG dosage increased, likely due to the relatively weaker RBSs of *crtZ*.

RBS engineering of *crtZ* has enhanced the production of glycosylated and total carotenoids by 25% and 72%, respectively, as compared to that of GA01. However, unlike the obvious positive effect of RBS of *crtX* on zeaxanthin glucosides (Fig. 3D), the data in Additional file 1: Figure S5 indicated the lack of correlation between the RBS strength of *crtZ* and carotenoid production. The lack of correlation was not surprising as the top two producers, GA03 and GA05, had relatively weaker RBSs.

## Discussion

Here, we successfully engineered *E. coli* to produce carotenoid glucosides in high amounts. Particularly, our zeaxanthin glucoside strain produced 11,670 ppm of two zeaxanthin glucosides (~ 7150 ppm of zeaxanthin diglucose, ~ 4520 ppm of zeaxanthin glucoside) in 2-day batch fermentation (Fig. 4B). In contrast, the astaxanthin glucoside strains (GA01–09) produced lower amount of total carotenoid glucosides (1774 ppm) but with high diversity where 7 carotenoid glucosides were detected. To the best of our knowledge, our study is the first to produce these carotenoid glucosides (up to 7 varieties) in recombinant microbes.

Glycosylation plays a crucial role in secondary metabolite biosynthesis such as carotenoids and flavonoids. Similar to carotenoids, the glycosylation improves their solubility, stability, and biological activities of flavonoids. The GTs for both flavonoids and carotenoids are GT1 family, for example, flavonol 3-O-glucosyltransferase (EC 2.4.1.91); anthocyanidin 3-O-glucosyltransferase (EC 2.4.1.115); ZGT (EC 2.4.1.-). The GT1 family comprises a highly divergent, polyphyletic genes/enzymes, with GTs identified from animals, plants, fungi, bacteria, and viruses. Flavonoid GTs are relatively well studied and characterized. To date, 35 flavonoid GTs are reviewed in UniProt database, and most of them show broad activities to a large range of structurally similar flavonoids and sugar donors, e.g., Anthocyanidin 3-O-glucosyltransferase UFGT from *Vitis vinifera* (UniProt ID, P51094) can accept cyanidin, delphinidin, kaempferol, malvidin, quercitin, etc. as substrates and use UDP-glucose, UDP-galactose, guanosine 5’-diphosphoglucose (GDP-glucose), dTDP-glucose, etc. as sugar donors. In contrast, none of the carotenoid GTs has been well studied. Our results here supported that ZGT was able to glycosylate various other carotenoids (e.g., adonirubin, adonixanthin), in addition to the reported zeaxanthin and astaxanthin (Hundle et al. 1992; Yokoyama et al. 1998). Furthermore, if xanthophylls have two hydroxyl groups (e.g., astaxanthin), diglycosylated products can also be produced by ZGT. Considering the complexity of the carotenoid pathway and the promiscuity of ZGT, the product diversity was not surprising as the glycosylation reaction competed with other reactions (hydroxylation or ketolation, Fig. 1). The presence of bulky glycoside moiety may prevent the glycosylated intermediates (e.g., zeaxanthin and adonixanthin) from further ketolation to astaxanthin glucosides by the β-carotene ketolase (*crtW*); hence, all the carotenoid glucosides became the end products (Fig. 1).

This study was largely built on our previous astaxanthin platform. By removing the *crtW* gene, we obtained a high-yield zeaxanthin strain, and furthermore, the high-yield production of zeaxanthin and astaxanthin glucosides. The success indicated that our carotenoid platform is highly expandable for the production of various carotenoids and serves a good starting point for further optimization. Yet even with such a good platform, it is still not trivial to further tune the pathways for the production of carotenoid glucosides, much efforts are still required to enhance the yields toward industrial viability. To improve the glycosylation of zeaxanthin, we have employed RBS engineering (strong RBS for ZGT), media optimization and supplementation of additional lycopene cyclase (*crtY*). All the strategies were very effective, collectively, they enhanced the yields of the two zeaxanthin glucosides from 1640 ppm to 11,670 ppm, or by 7.1 fold.

However, it was not straightforward for astaxanthin glycosylation. A possible reason is that the ZGT from *P. ananatis* might have relatively lower activity for astaxanthin than zeaxanthin. The keto group may also stabilize the hydroxyl group or introduces steric hindrance and, thus, reduces accessibility by ZGT. Also, the competitions for carotenoid intermediates by ketolases (CrtW), hydroxylases (CrtZ) and ZGT increase the ramification of the metabolic pathway. To further improve the production of astaxanthin glucosides, four strategies can be employed in the future: (1) to explore the natural diversity of ZGTs for more suitable enzymes; (2) to balance the expression of Module 4 (Fig. 1); (3) to further manipulate the intracellular UDP-glucose supply; and 4) to implement a dynamic regulation to trigger glycosylation after the formation of astaxanthin. A search in UniProt database resulted in 254 zeaxanthin GT homologues from 69 microbial genera, particularly in *Pseudomonas*, *Pantoea* and *Massilia*, which have 88, 22, 12 of homologues identified, respectively. Experimental screening may lead to identifying some candidates with higher activities and/or specificities for astaxanthin. Furthermore, the data in Figs. 3 and 6 indicated that the perturbation of *crtZ* and *crtX* expression had strong effects on both yields of total carotenoids and glycosylated carotenoids. The parental strain (Ast) had produced astaxanthin as the main product; however, all the GA01–09 strains had β-carotene accumulated intracellularly (Fig. 6B). This indicated that previously balanced pathway was perturbed by the introduction of ZGT. A solution is to refine the module 4 by RBS/promoter engineering or organization shuffling of operon genes to minimizing the accumulation of intermediates (e.g., lycopene and β-carotene, Fig. 6B). Lastly, unlike zeaxanthin glycosylation strain with high glycosylation efficiency (> 90%), the astaxanthin glycosylation was relatively low (40–50%) indicating they might be still limited by the accessible intracellular UDP-glucose, whose supply can be enhanced by overexpressing UDP-glucose biosynthetic pathway genes (e.g., *glk*: glucokinase, *pgm*: phosphoglucomutase, *galU*: UDP-glucose pyrophosphorylase) and by utilizing other types of UDP-sugars with glycosyltransferases. The strategy has been successfully applied to increase the production of flavonoids such as anthocyanins (Shrestha et al. 2019; Zha et al. 2020) and is worth exploring on carotenoid glycosylation.

## Conclusion

We have developed microbial strains to overproduce various carotenoid glucosides. The metabolic engineering and bioprocess strategies are proven to be effective and have synergic effects in improving the yields of carotenoid glucosides by balancing the metabolic pathways and supplying carbon precursors and important cofactors. Our study here demonstrated a proof-of-concept study for microbial production of glycosylated carotenoids and might inspire the production for other high-value metabolites, especially other glycosylated metabolites.

## Methods

### Strain and plasmid construction

*E. coli Bl21-Gold DE3 strain (Stratagene)* was used in this study. The plasmids p15A-*spec-hmgS-atoB-hmgR* (L2-8), p15A-*spec-crtY-hmgS-atoB-hmgR* (L2-8) p15A-*cam-mevK-pmk-pmd-idi* (L2-5), p15A-*kan-crtEBI-ispA* were designed as previously described (Zhang et al. 2018). The zeaxanthin GT gene *(crtX)* from *Pantoea ananatis* was codon optimized (DNA sequence was provided in Additional file 1: Supplementary note) and synthesized by Integrated DNA Technologies, Singapore. Subsequently, crtX was cloned with the primers (Additional file 1: Table S2) into the operon of the plasmids p15A-amp-crtYZ (L2-9) and p15A-amp-crtYZW (L2-9) (Zhang et al. 2018) to obtain p15A-amp-crtYZX and p15A-amp-crtYZWX, respectively.

### Construction of RBS library

CrtZ RBS library was created using the degenerate primer and followed by screening and sequencing validations, using the same cloning method as previously described (Zhang et al. 2018). RBS strengths or translation efficiencies were predicted by RBS Calculator, version 2.0 (Farasat et al. 2014).

### Tube culture of the *E. coli* strains

The medium used was TB medium (20 g/L tryptone, 24 g/L yeast extract, 17 mM KH_2_PO_4_, and 72 mM K_2_HPO_4_) and 2XPY medium (20 g/L peptone, 10 g/L yeast extract and 10 g/L NaCl), supplemented with 10 g/L glycerol or 10–20 g/L glucose or their mixture (5 g/L glucose + 5 g/L glycerol), 50 mM 4-(2-hydroxyethyl)-1-piperazineethanesulfonic acid (HEPES), as previously described (Zhang et al. 2018). For strain optimization, the cells were grown in 1 mL of TB or 2XPY medium in 14 ml BD Falcon™ tube at 28 °C/250 rpm for 2–3 days. The cells were also grown in 50 mL culture in shaking flasks for validation of the carotenoid production. The cells were initially grown at 37 °C/250 rpm until OD_600_ reached ~ 0.8, induced by 0.03–0.1 mM IPTG, and were subsequently grown at 28 °C for 2 days. The antibiotics (34 μg/ml chloramphenicol, 50 μg/ml kanamycin, 50 μg/ml spectinomycin and 100 μg/ml ampicillin) were supplemented in the culture to maintain the four plasmids.

### Microscope imaging of *E. coli* cells

For microscopy assay, *E. coli* cells were directly sampled from cell cultures. Cell amount was normalized by OD_600_ and directly observed at 1000 magnitude using a Leica DM6000B microscope. Neither centrifuge nor washing steps were introduced to avoid perturbation of the cell morphologies.

### Extraction and quantification of carotenoids

Total intracellular carotenoids were extracted from cellular pellets according to the acetone extraction method (Zhang et al. 2018). Briefly, 10–50 µL bacterial culture (depending on the content of carotenoids in the cells) was collected and centrifuged. Cell pellets were washed with PBS and were resuspended in 20 µL of water, followed by addition of 180 µL of acetone and vigorous homogenization for 20 min. After 10 min of centrifugation at 14,000 g, the supernatant was collected and filtered using a PTFE, 0.45 μm filter.

The separation of carotenoids from cytosol and cell membranes was done by differential centrifugation. Briefly, cell pellets collected from 1 mL of culture were resuspended in 1 ml lysis buffer (50 mM Tris HCl of pH 7.5, 200 mM NaCl, 1 mg/ml lysozyme of pH 8) before 3 × 30 s sonication at 4 °C (75% amplitude). The cell lysate was subsequently centrifugated for 10 min at 14,000 g. The supernatant containing the cytosol fraction of carotenoids and the pellet debris containing the membrane fraction were extracted separately with by 1 mL of extraction buffer (hexane: acetone: ethanol at 2:1:1 volumetric ratio).

### Quantification of carotenoids

All the carotenoids were analyzed by Agilent 1290 Infinity II UHPLC System coupled with Diode Array Detector (DAD) detector and 6230B TOF-MS platform. The LC/MS method was similar to previously described (Zhang et al. 2018). Briefly, 1 μL of purified carotenoids in acetone was injected into the Agilent ZORBAX RRHD Eclipse Plus C18 2.1X50 mm, 1.8 um. Separation was carried out at a flow rate of 0.5 mL/min. The mobile phase and gradient used were as follows. The analysis started from 10% water (0.1% formic acid), 10% methanol (0.1% formic acid) and 80% acetonitrile (0.1% formic acid) and this condition was maintained for 2 min, followed by the increase in methanol from 10 to 90% and the decrease in water from 10% to 0 and acetonitrile from 80 to 10% within 0.1 min. The condition (90% methanol and 10% acetonitrile) was continued for 7 min. The whole analysis finished at 10 min. Mass spectrometry was operated to scan 100–1100 m/z in ESI-positive mode with 4000 V capillary voltage. Nebulizer gas was supplied at 35 psig and dry gas flow was 10 L/min. Gas temperature was set at 325 °C. Shealth gas was set at 350 °C and 12 L/min. Retention time was determined with chemical standards or calculated based on chromatography profile for those carotenoids without standards.

Carotenoid concentrations were calculated based on the peak area of each compound extracted by their corresponding m/z value (Table 1) or UV absorbance at 450 nm (Additional file 1: Figure S2). Standard curves were generated for the five chemical standards with extracted-ion chromatogram (EIC) peak areas (Additional file 1: Figure S3): lycopene, β-carotene, astaxanthin, canthaxanthin (Sigma-Aldrich, St. Luis, MO, USA), and zeaxanthin (Santa Cruz Biotechnology, Dallas, TX, USA). For those carotenoids without standards, the concentration was calculated based on the relative peak area to its close compartment. For example, the concentrations of zeaxanthin glucoside and zeaxanthin diglucoside were calculated based on that of zeaxanthin; the concentrations of astaxanthin glucosides, adonixanthin and its diglucosides were calculated based on that of astaxanthin. Carotenoid contents were calculated by normalizing the titres with dry cell weight (µg carotenoids per gram DCW, or ppm) (Zhang et al. 2018).

### Supplementary Information

**Additional file 1:** Supplementary Note. **Table S1.** Strains and their RBSs. **Table S2.** Primers used in this study. **Figure S1.** The UPLC chromatograms of UV (DAD). Five carotenoids were detected: 1 -zeaxanthin-β-D-diglucoside, 2 - zeaxanthin-β-D-glucoside, 3 - zeaxanthin, 4 - lycopene, 5 - β-carotene. **Figure S2.** The mass spectra of various carotenoids detected. **Figure S3.** UPLC chromatograms of UV (DAD) and extracted-ion monitoring (EIC) of five standards. 1 - astaxanthin; 2 - zeaxanthin; 3 - canthaxanthin; 4 - lycopene; and 5 - β-carotene. **Figure S4.** LC/MS chromatograms of various carotenoids. 3'-hydroxyechinenone, β-cryptoxanthin-β-D-glucoside and 3'-hydroxyechinenone-β-D-glucoside were not detected (n.d.) in none of the nine strains GA01-09. **Figure S5.** Correction of RBS strength with the yields of different carotenoids and OD_600_.

## Data Availability

All data supporting the findings of this study are available in the article, Additional file 1, or upon request from the corresponding author.

## Abbreviations

GT: Glycosyltransferase
UDP-glucose: Uridine diphosphate-α-D-glucose
ZGT: Zeaxanthin glucosyltransferase
IPTG: Isopropyl β-d-1-thiogalactopyranoside
RBS: Ribosomal-binding site
