# Supplementary material for: High-level de novo biosynthesis of glycosylated zeaxanthin and astaxanthin in Escherichia coli
Source: Bioresour Bioprocess. 2021 Jul 29;8(1):67. doi: 10.1186/s40643-021-00415-0 (PMC10992962; doi:10.1186/s40643-021-00415-0)
Supplement: Supplementary file 1 — Additional file 1: Supplementary Note. Table S1. Strains and their RBSs. Table S2. Primers used in this study. Figure S1. The UPLC chromatograms of UV (DAD). Five carotenoids were detected: 1 -zeaxanthin-β-D-diglucoside, 2 - zeaxanthin-β-D-glucoside, 3 - zeaxanthin, 4 - lycopene, 5 - β-carotene. Figure S2. The mass spectra of various carotenoids detected. Figure S3. UPLC chromatograms of UV (DAD) and extracted-ion monitoring (EIC) of five standards. 1 - astaxanthin; 2 - zeaxanthin; 3 - canthaxanthin; 4 - lycopene; and 5 - β-carotene. Figure S4. LC/MS chromatograms of various carotenoids. 3'-hydroxyechinenone, β-cryptoxanthin-β-D-glucoside and 3'-hydroxyechinenone-β-D-glucoside were not detected (n.d.) in none of the nine strains GA01-09. Figure S5. Correction of RBS strength with the yields of different carotenoids and OD600. [file 40643_2021_415_MOESM1_ESM.pdf]

De novo biosynthesis of glycosylated carotenoids in *Escherichia coli*

Xixian Chen[1], Xiao Hui Lim[1], Aurelie Bouin[1,2], Thomas Lautier[1,2], Congqiang Zhang[1*]

[1]*Singapore Institute of Food and Biotechnology Innovation (SIFBI), Agency for Science, Technology and Research (A*STAR), Singapore.*

[2]*TBI, Université de Toulouse, CNRS, INRAE, INSA, Toulouse, France*

* To whom correspondence should be addressed.

Congqiang Zhang**:** SIFBI, A*STAR, Proteos level 4, Singapore 138673;

Email: zcqsimon@outlook.com; congqiang_zhang@sifbi.a-star.edu.sg

Supplementary Note:

crtX codon-optimized sequence in this study:
atgtctcacttcgcggcgattgctcctccgttctacagccacgttcgtgcgctgcagaatttggctcaggaattggtagcgcgtggtcatcgcgtgac
cttcatccagcaacacgacattaaacatctgattgactctgaaaccatcggattccacgctgtaggtactgattctcatccaccggctcgcctcacc
cgtgtactgcatctggcagcacacccgctgggtccgtctatgctgaaactgattaacgagatggcacgcactaccgatatgctgtgtcgcgaactg
ccgcgtgcgttcaacgatttggcggttgatggcgtcatcgttgaccaaatggaaccggccggcgcgctggttgctgaggctctgggcctgccgttta
tctccgtagcttgcgcgctgccgttaaaccgtgagccggacatgccgttggcggtaatgccgtttgaatacggaacttctgacgcagcgcgcgaa
cgttacgcagcctctgaaaaaatttacgactggctgatgcgtcgtcacgaccgcgttatcgcggaacactcgcaccgcatggggctggcgccgc
gtcagaagctgcaccagtgcttctctccgctggcgcaaatcagccagctggtaccggaactggattttccgcgcaaagctctgccggcttgtttcca
cgcggttgggccactgcgtgaaacttatgctccgtctacgagctcgtcccgctatttcacttcttcggagaaaccgcgtatttttgcaagtctgggcac
cctgcagggccatcgctacggtctgtttaaaacaattgttaaagcgtgtgaagaaattgacggccaactgctgctggcgcattgcggtcgcctgac
cgattctcaatctgaagaactggctcgctctcgtcatacccaggttgtagatttcgccgatcagagcgcggcgctgagccaggcgcagctggcga
tcacccacggtggcatgaacactgttctggatgctattaactaccgcaccccgttgctggcactgccgctggcattcgaccagcctggtgttgcgag
ccgcattgtgtatcacggcattggtaaacgtgcatcgcgcttcaccacgtcccacgcactggctcgccagatgcgctccctgctgaccaacctgg
acttccagcagcgtatggcgaaaatccagaccgccctgcgtctggccggcggtaccatggctgcagcggacattatcgaacaagttatgcgtac
cggccagccagttctgagcggtagcggttatgctaccgccctgtga

Tables

Supplementary Table S1. Strains and their RBSs.

Strain names	crtZ RBS sequence	RBS strength	Relative strength
Consensus	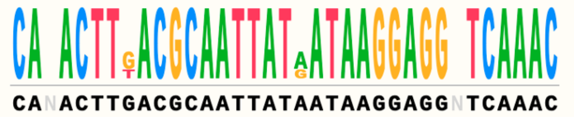	/	/
GA01	CAAACTTGACGCAATTATAATAAGGAGGTTCAAAC	43498	1.00
GA02	CAAACTTTACGCAATTATAATAAGGAGGTTCAAAC	29805	0.69
GA03	CAAACTTGACGCAATTATAATAAGGAGGGTCAAAC	2085	0.05
GA04	CATACTTGACGCAATTATAATAAGGAGGATCAAAC	7473	0.17
GA05	CATACTTGACGCAATTATGATAAGGAGGTTCAAAC	4355	0.10
GA06	CAAACTTTACGCAATTATAATAAGGAGGGTCAAAC	2858	0.07
GA07	CATACTTTACGCAATTATAATAAGGAGGATCAAAC	29538	0.68
GA08	CAGACTTGACGCAATTATGATAAGGAGGGTCAAAC	2973	0.07
GA09	CATACTTGACGCAATTATGATAAGGAGGGTCAAAC	585	0.01

Supplementary Table S2. Primers used in this study.

Primers	Sequence
I-p15A-crtYZ(-)-F	AAGGA*AGCTG*AGTTGGCTG
I-p15A-crtYZ(-)-R	TTACT*TACCA*GATGCCGGTT
I-crtX(YZ-R)-F	TGGTA*AGTAA*TATCCGCACCCAATTCACT
I-crtX(YZ-F)-R	CAGCT*TCCTT*TCACAGGGCGGTAGCATA

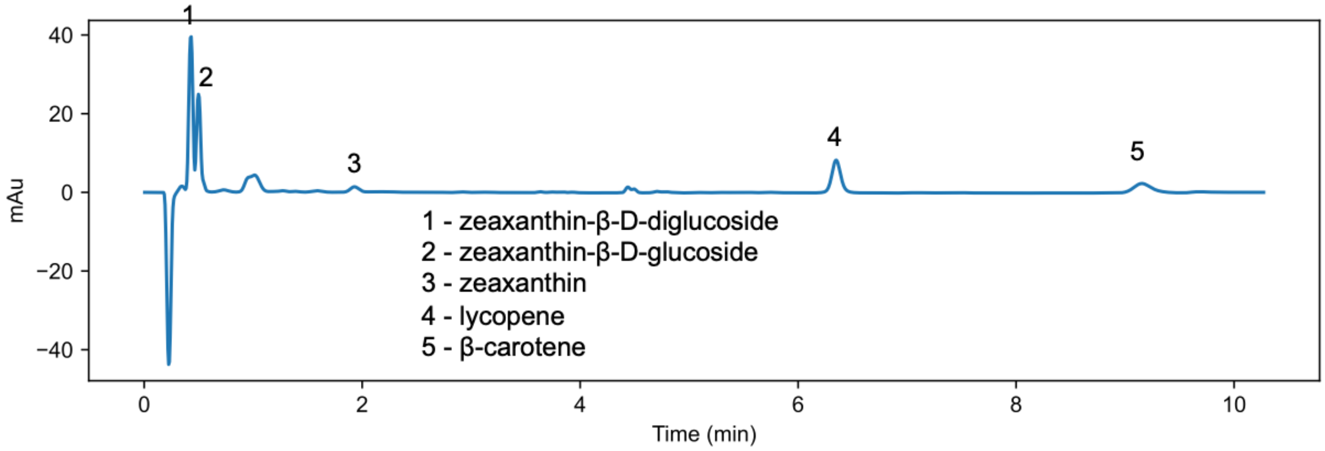

Supplementary Figure S1. The UPLC chromatograms of UV (DAD). Five carotenoids were detected: 1 - zeaxanthin-β-D-diglucoside, 2 - zeaxanthin-β-D-glucoside, 3 - zeaxanthin, 4 - lycopene, 5 - β-carotene.

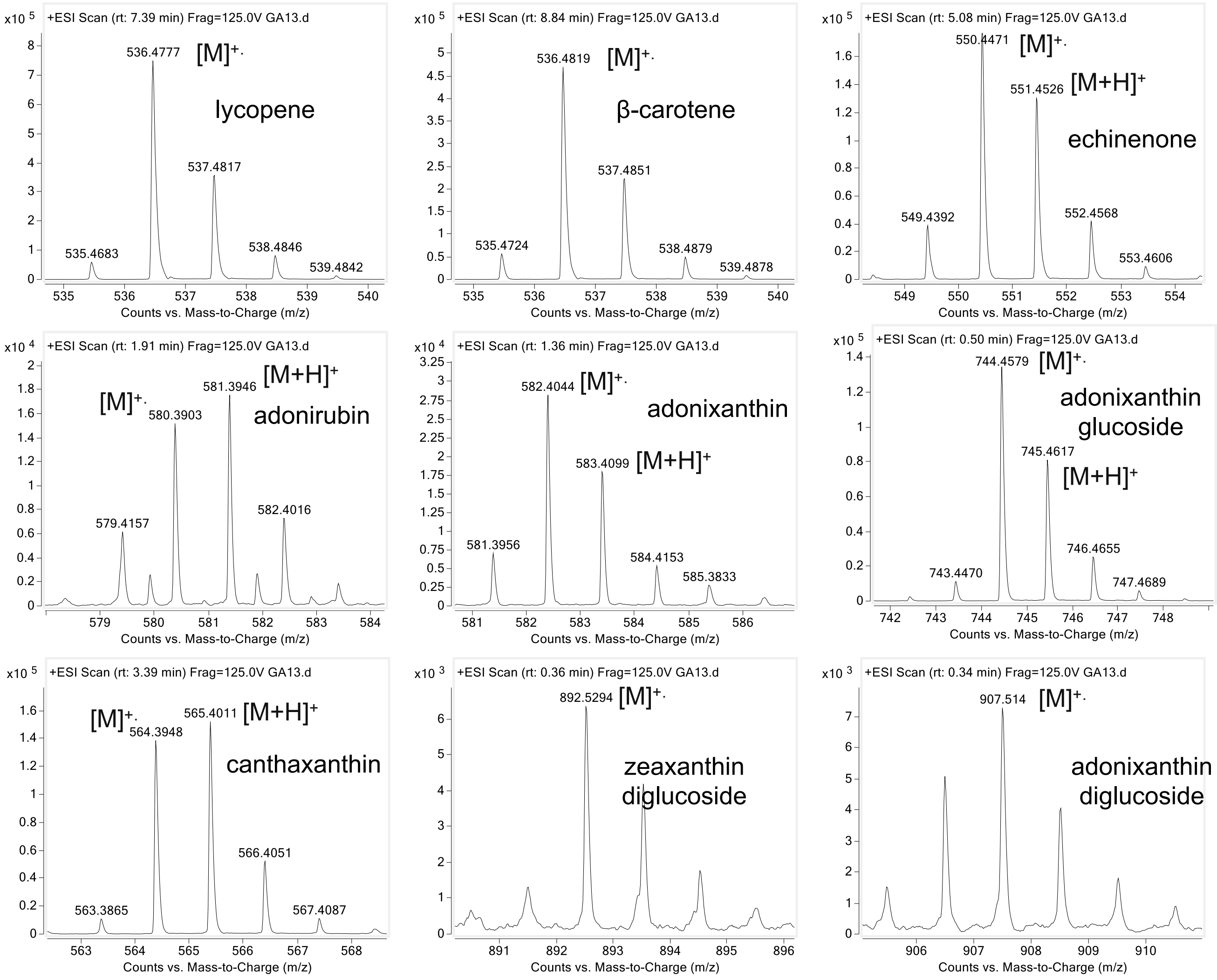

Supplementary Figure S2. The mass spectra of various carotenoids detected.

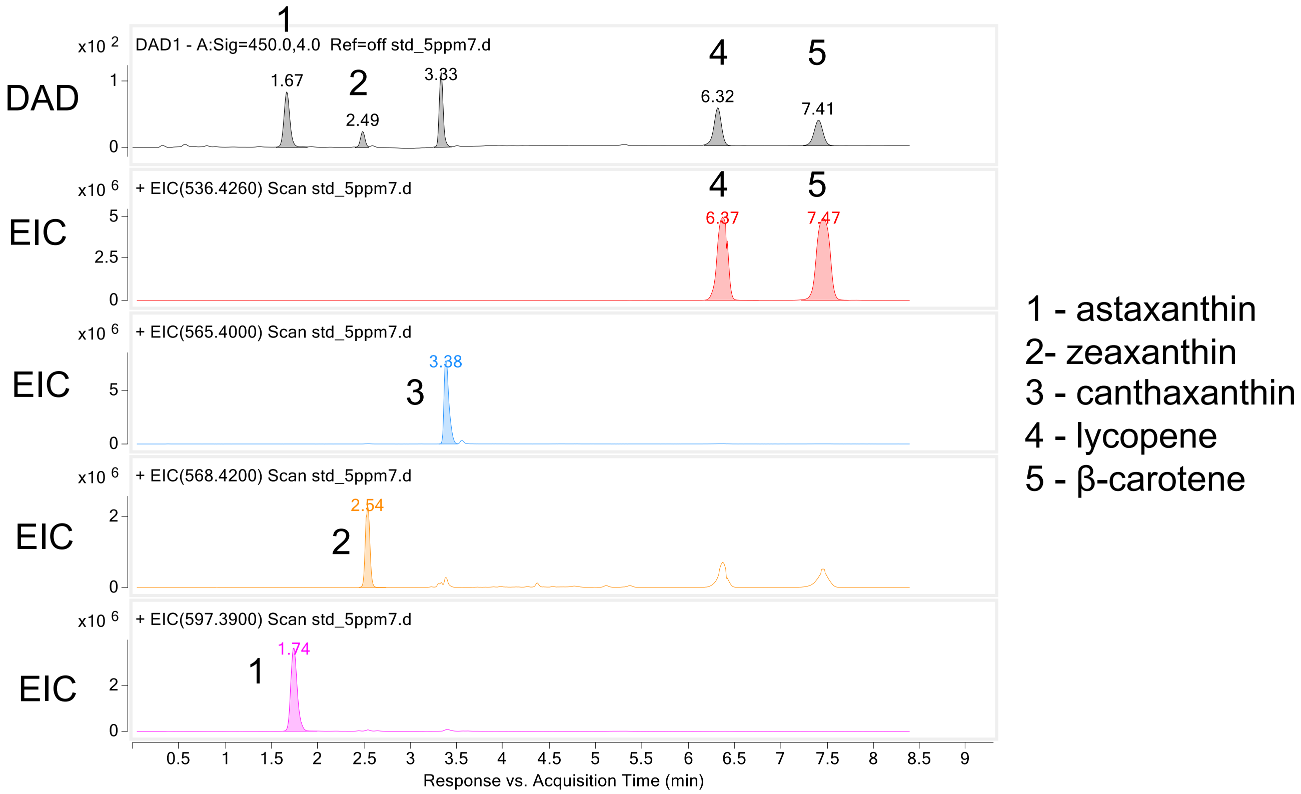

Supplementary Figure S3. UPLC chromatograms of UV (DAD) and extracted-ion monitoring (EIC) of five standards.

1 - astaxanthin; 2 - zeaxanthin; 3 - canthaxanthin; 4 - lycopene; and 5 - β-carotene

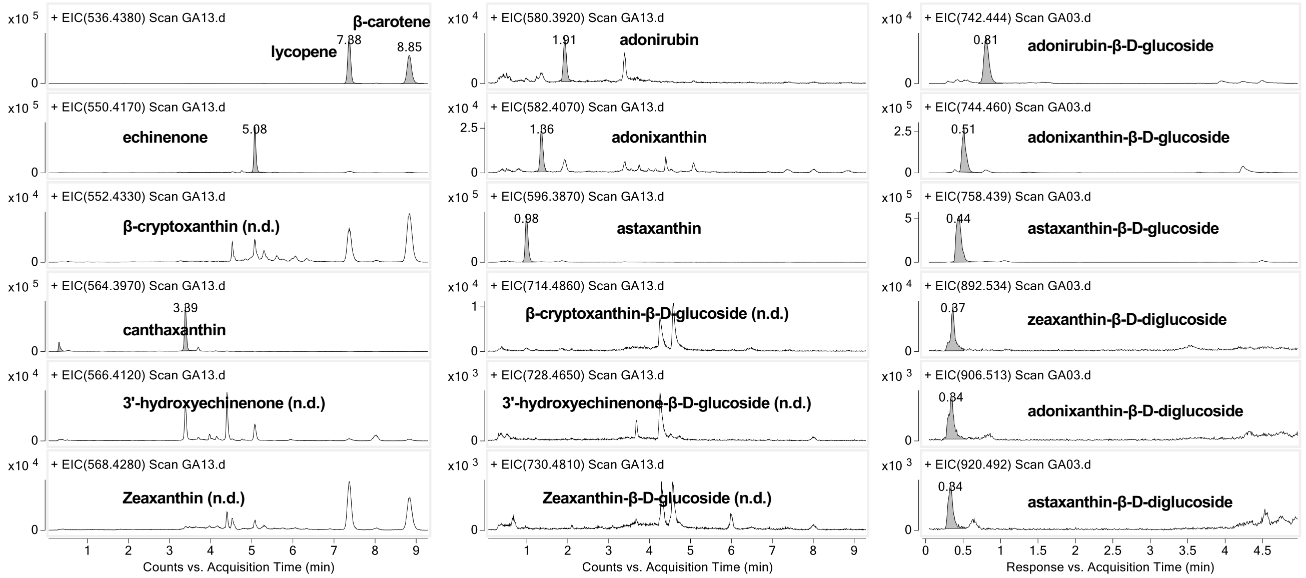

Supplementary Figure S4. LC/MS chromatograms of various carotenoids.

3'-hydroxyechinenone, β-cryptoxanthin-β-D-glucoside and 3'-hydroxyechinenone-β-D-glucoside were not detected (n.d.) in none of the nine strains GA01-09.

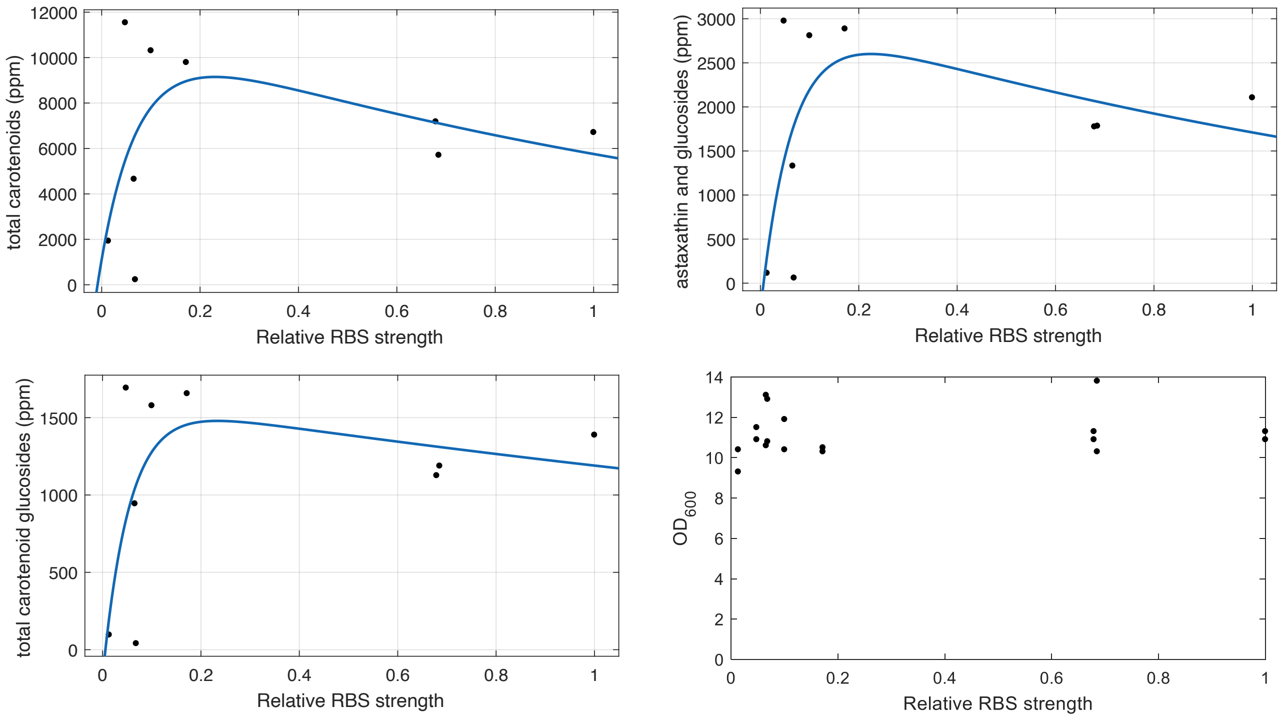

Supplementary Figure S5. Correction of RBS strength with the yields of different carotenoids and OD_{600}.
